# Evaluation of AT-752, a Double Prodrug of a Guanosine Nucleotide Analog with *In Vitro* and *In Vivo* Activity against Dengue and Other Flaviviruses

**DOI:** 10.1128/AAC.00988-21

**Published:** 2021-10-18

**Authors:** Steven S. Good, Ashleigh Shannon, Kai Lin, Adel Moussa, Justin G. Julander, Paolo La Colla, Gabriella Collu, Bruno Canard, Jean-Pierre Sommadossi

**Affiliations:** a Atea Pharmaceuticals, Inc., Boston, Massachusetts, USA; b Architecture et Fonction des Macromolécules Biologiques, Marseille, France; c Institute for Antiviral Research, Utah State University, Logan, Utah, USA; d Dipartimento di Scienze e Tecnologie Biomediche, Università degli Studi di Cagliari, Monserrato, Italy

**Keywords:** AT-752, AT-281, AT-9010, dengue, flavivirus, PBMCs, antivirals, virology

## Abstract

Every year, millions of people worldwide are infected with dengue virus (DENV), with a significant number developing severe life-threatening disease. There are currently no broadly indicated vaccines or therapeutics available for treatment of DENV infection. Here, we show that AT-281, the free base of AT-752, an orally available double prodrug of a guanosine nucleotide analog, was a potent inhibitor of DENV serotypes 2 and 3 *in vitro*, requiring concentrations of 0.48 and 0.77 μM, respectively, to inhibit viral replication by 50% (EC_50_) in Huh-7 cells. AT-281 was also a potent inhibitor of all other flaviviruses tested, with EC_50_ values ranging from 0.19 to 1.41 μM. Little to no cytotoxicity was observed for AT-281 at concentrations up to 170 μM. After oral administration of AT-752, substantial levels of the active triphosphate metabolite AT-9010 were formed *in vivo* in peripheral blood mononuclear cells of mice, rats, and monkeys. Furthermore, AT-9010 competed with GTP in RNA template-primer elongation assays with DENV2 RNA polymerase, which is essential for viral replication, with incorporation of AT-9010 resulting in termination of RNA synthesis. In AG129 mice infected with DENV D2Y98P, treatment with AT-752 significantly reduced viremia and morbidity and increased survival. The demonstrated *in vitro* and *in vivo* activity of AT-752 suggests that it is a promising compound for the treatment of dengue virus infection and is currently under evaluation in clinical studies.

## INTRODUCTION

Flaviviruses are single-stranded positive-sense RNA viruses that include the mosquito-borne dengue virus (DENV), West Nile virus (WNV), Japanese encephalitis virus (JEV), Powassan virus (POWV), Usutu virus (USUV), yellow fever virus (YFV), and Zika virus (ZV). According to the Centers for Disease Control and Prevention (https://www.cdc.gov/dengue/about/index.html), among these viruses, DENV produces the highest incidence of illness, with an estimated 400 million infections each year, of which approximately 100 million result in clinical manifestations, including dengue hemorrhagic fever, or severe dengue, and approximately 25,000 deaths. The recent decades have witnessed a drastic resurgence of DENV with a >8-fold increase of dengue cases reported to the World Health Organization (WHO) (https://www.who.int/news-room/fact-sheets/detail/dengue-and-severe-dengue) in the past 20 years. Large outbreaks, while mostly in tropical and subtropical regions, including Africa, Southeast Asia, and South America, have also occurred in parts of Europe (1, 2), and dengue is now endemic in more than 100 countries/regions worldwide (3). Dengue fever is also on the rise in the United States, with over 5,000 cases, mostly travel associated, reported between 2010 and 2017 (4). There are four antigenically distinct but closely related DENV serotypes (DENV1 to 4). Isolation of a fifth serotype (DENV5) was reported in 2015 but has yet to be officially recognized (5). The WHO (https://www.who.int/news-room/fact-sheets/detail/dengue-and-severe-dengue) states that recovery from infection is believed to provide lifelong immunity against that specific serotype; however, cross-immunity to the other serotypes after recovery is only partial and transient. Furthermore, subsequent infection by other serotypes increases the risk of developing severe dengue through a process known as antibody-dependent enhancement (ADE), which, in turn, increases the risk of death.

There are no direct-acting antivirals (DAAs) or broadly indicated vaccines for DENV. The only licensed vaccine, Dengvaxia, offers incomplete (https://www.fda.gov/news-events/press-announcements/first-fda-approved-vaccine-prevention-dengue-disease-endemic-regions) and uneven protection against different serotypes, with only 50% protection against DENV1 and less for DENV2 (6, 7). Vaccination can therefore promote ADE and, consequently, severe infection in dengue-naive individuals (8) and thus is restricted to individuals 9 to 16 years of age who have had at least one documented previous dengue virus infection. Viral polymerases, critical enzymes in viral replication, have proven to be valid targets for DAAs to inhibit RNA and DNA viruses, including hepatitis B and C viruses (9). In DENV, the nonstructural protein 5 (NS5) is such an enzyme, functioning as the viral RNA-dependent RNA polymerase (RdRp) (10). Its highly conserved nature potentially allows for a single agent to be active against all serotypes. Although the DENV RdRp has been the target of many investigational DAAs, including many nucleoside/nucleotide analogues (11), to date, only one nucleoside, the cytosine analog balapiravir, has been tested clinically (10, 12). Unfortunately, no differences between compound and placebo treatment were observed regarding antiviral response, cytokine profile, and time to fever clearance (12).

**FIG 1:**
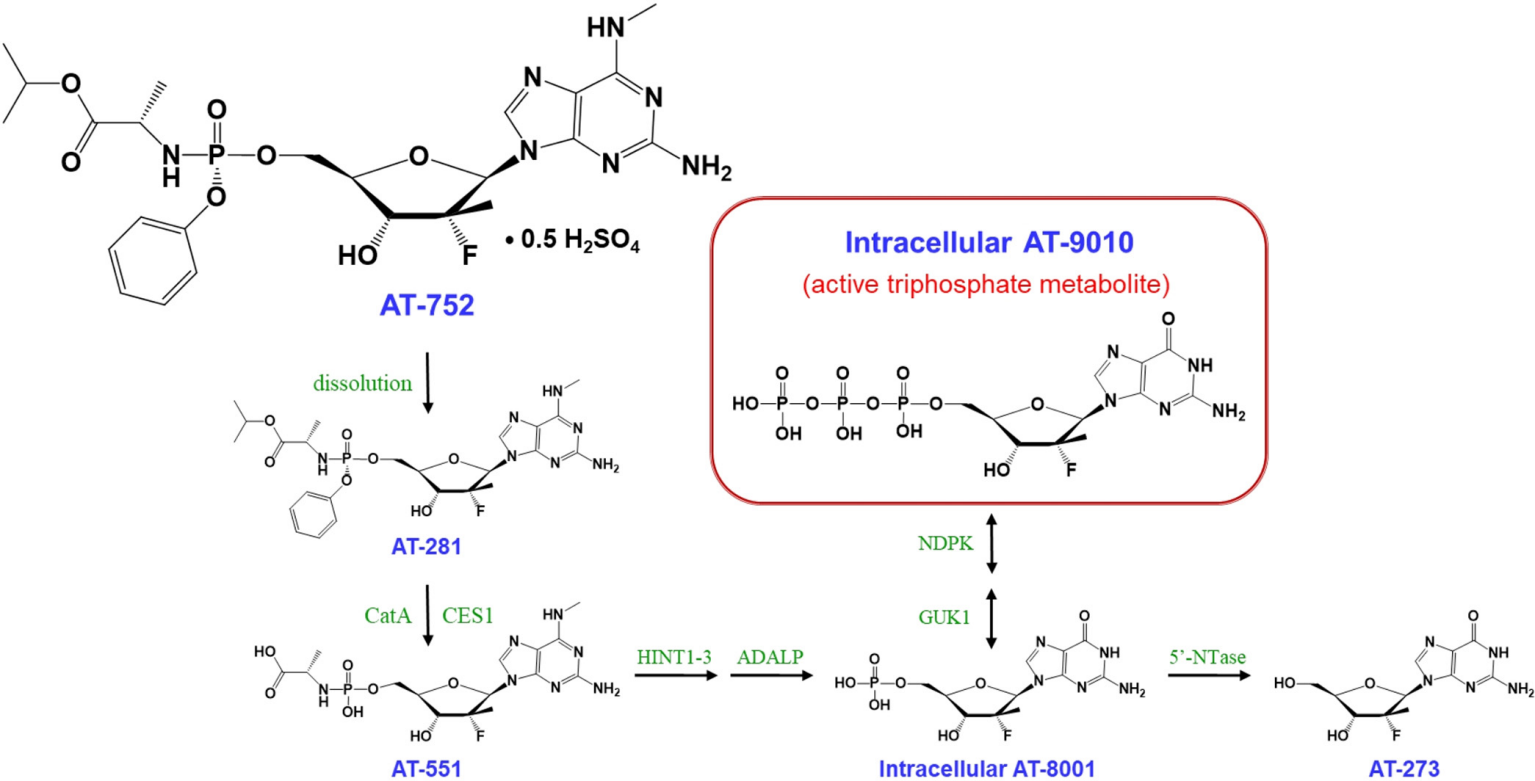
AT-752 and its putative metabolic pathway to the pharmacologically active metabolite AT-9010.

Here, we report that AT-752, an orally available double prodrug of a guanosine nucleotide analog, has potent *in vitro* activity against DENV2 and DENV3, as well as all other flaviviruses tested, and has demonstrated *in vivo* activity against DENV2 in a mouse model of disease. AT-752 is the hemisulfate salt of AT-281, a phosphoramidate protide which forms the l-alanyl metabolite AT-551 as an intermediate prodrug before being converted to the 5′-monophosphate (MP) metabolite (AT-8001) of the nucleoside 2′-fluoro-2′-C-methyl guanosine (AT-273) (Fig. 1). AT-8001 is then phosphorylated to the active 5′-triphosphate (TP) metabolite (AT-9010). AT-281 is a diastereomer of its congener AT-511, whose hemisulfate salt AT-527 is under clinical development for treatment of coronavirus disease 2019 (COVID-19) (13) and hepatitis C virus (HCV) infection (14, 15). The only structural difference is that AT-281 is the Rp-diastereomer, whereas AT-511 is the Sp-diastereomer. Because of their structural similarity, the two compounds are presumed to be subject to the same metabolic activation pathway resulting in AT-9010, the intracellular antivirally active TP, and its plasma surrogate nucleoside marker AT-273 (15). AT-9010 has been shown to selectively inhibit the viral RNA-dependent RNA polymerase (RdRp) of HCV (15), and here we show that it appears to inhibit NS5, the RdRp of DENV2. Given that the RdRp active site is highly conserved in *Flaviviridae* (16–18), AT-9010 may be effective against all DENV serotypes, which makes AT-752 an attractive candidate for clinical development.

## RESULTS

### AT-752 inhibits DENV and other flaviviruses.

To assess whether AT-281, the free base of AT-752, had antiviral activity against DENV and other flaviviruses, human hepatocarcinoma (Huh-7) cells, baby hamster kidney (BHK-21) cells, or human peripheral blood mononuclear cells (PBMCs) were acutely infected with the different viruses and exposed to serial dilutions of the drug. After a 3- to 6-day incubation, the effective concentration of AT-281, a 2′-fluoro-2′-C-methyl guanosine nucleotide prodrug, required to achieve 50% inhibition (EC_50_) of the virus-induced cytopathic effect (CPE) ranged from 0.21 to 1.41 μM (Table 1). While the growth kinetics of different flaviviruses can differ in cell culture, AT-281 showed similar potent effects in reducing viral titers against all 7 flaviviruses, including 11 different strains tested *in vitro*. The inhibition curves for DENV, WNV, and YFV (see Fig. S1 in the supplemental material) demonstrate this consistency. The concentration of AT-281 required to kill 50% of cells exposed to drug only (CC_50_) exceeded the highest concentration tested (85 to 170 μM), and the antiviral selectivity index (SI; CC_50_/EC_50_) ranged from >120 to >650. In particular, AT-281 exhibited potent multiserotypic anti-DENV activity *in vitro*. In Huh-7 cells infected with DENV2 (New Guinea C strain), AT-281 reduced the yield of infectious virus by 90% (EC_90_) at a concentration of 0.64 μM. Similarly, in human PBMCs and BHK-21 cells infected with DENV2 (clinical isolate) and in Huh-7 cells infected with DENV3 (H87 strain), AT-281 exhibited respective EC_50_ values of 0.60, 0.63, and 0.77 μM in inhibiting virus-induced CPE. The SI was >210 in each case.

**TABLE 1:** Antiviral activity of AT-281 in cells infected with various flaviviruses^*a*^

Virus	Strain	Host cell	EC_50_^*b*^ (μM)	EC_90_^*c*^ (μM)	CC_50_^*d*^ (μM)
Dengue-2	New Guinea C	Huh-7	0.48	0.64	>170
	Clinical isolate	Human PBMC	0.80	ND^*e*^	>85
	Clinical isolate	BHK-21	1.1	ND	>170
Dengue-3	H87	Huh-7	0.77	ND	>170
Japanese encephalitis	SA-14	Huh-7	0.21	ND	>170
West Nile	Kern 515, WNo2	Huh-7	1.41	0.43	>170
Yellow Fever	YFV 17D	Huh-7	0.31	0.26	>170
Zika	MR766	Huh-7	0.64	ND	>170
Powassan	Spooner	BHK-21	0.29	0.74	>170
	LB	BHK-21	0.64	2.6	>170
Usutu	TC-508	Huh-7	0.19	0.72	>170

a The activity of AT-281 was measured in infected cells using the neutral red assay and/or the virus yield reduction (VYR) assay as described in Materials and Methods to determine the effective concentration required to achieve 50% inhibition (EC_50_) of the virus-induced cytopathic effect (CPE), the concentration to reduce virus yield by 1 log_10_ (EC_90_), and the cytotoxic concentration of the drug to cause death to 50% of viable cells incubated without virus (CC_50_).

b Neutral red assay.

c VYR assay.

d Highest concentration tested.

e ND, not determined.

### *In vitro* antiviral specificity of AT-281.

To assess the antiviral specificity of AT-281, serial dilutions were incubated with various host cell lines infected with a panel of DNA and RNA viruses other than flaviviruses. AT-281 had no activity against the DNA viruses tested and was only weakly or not active against several RNA viruses (Table 2). However, it had a high selectivity (>2,000) toward HCV, inhibiting the virus at nanomolar levels (Table 2).

**TABLE 2:** Antiviral selectivity of AT-281 against a panel of RNA and DNA viruses^*a*^

Virus	Type	Cell type	EC_50_ (μM)	CC_50_ (μM)
Chikungunya	RNA	Huh-7	54.2	>100
Eastern equine encephalitis	RNA	Huh-7	10.2	>100
HCV	RNA	Huh-7	0.05	>100
HIV-1	RNA	CEM-SS	>100	>100
Influenza A and B	RNA	MDCK	>100	>100
Respiratory syncytial virus	RNA	HEp2	>100	>100
Rhinovirus	RNA	H1-HeLa	>100	>100
Rift Valley fever	RNA	Huh-7	>100	>100
Adenovirus	DNA	Vero	>100	>100
Herpes simplex-1	DNA	Vero	>100	>100

a The activity of AT-281 was measured in infected cells using assays previously described (see Materials and Methods and reference 15) to determine the effective concentration required to inhibit viral replication 50% (EC_50_) and the threshold concentration required to obtain a perceptible effect on 50% of the cells incubated without virus (CC_50_).

The potential cytotoxicity of AT-281 was assessed in multiple cell lines, with resulting CC_50_ values greater than 100 μM (Table 2). A further lack of cytotoxicity was demonstrated in human-induced pluripotent stem cell (iPS) cardiomyocytes and in granulocyte macrophage (GM) and erythroid (E) human bone marrow progenitor cells. When incubated with AT-281, these cells also had CC_50_ values greater than 100 μM, whereas the positive-control compounds had the expected cytotoxic effects (CC_50_ values of 7 μM for doxazosin with cardiomyocytes and 2 and 3 μM for zidovudine (AZT) in the GM and E cell assays, respectively). Additionally, we have previously shown that the active TP, AT-9010, does not inhibit the *in vitro* enzyme activities of human cellular DNA-dependent DNA polymerases α, β, or γ, with estimated 50% inhibitory concentration (IC_50_) values of >100 μM, and is it not likely to affect mitochondrial integrity or inhibit human mitochondrial DNA-directed RNA polymerase (POLRMT) (15).

### AT-281 forms the active metabolite AT-9010 in human PBMCs *in vitro*.

Since virus infection in the blood is an important component of dengue fever, it was important to determine the stability of AT-281 in blood and the ability of PBMCs to phosphorylate AT-281 to form the active TP metabolite, AT-9010. AT-752 was found to be stable for up to 120 min in cynomolgus monkey and human plasma (98.2% and 93.5% of the respective starting 2 μM concentrations remained after 120 min of incubation) but was highly unstable in Sprague Dawley rat plasma, with no drug remaining at the first time point (10 min of incubation). The half-life (*t*_1/2_) in rat plasma at 37°C was estimated to be <3 min.

**FIG 2:**
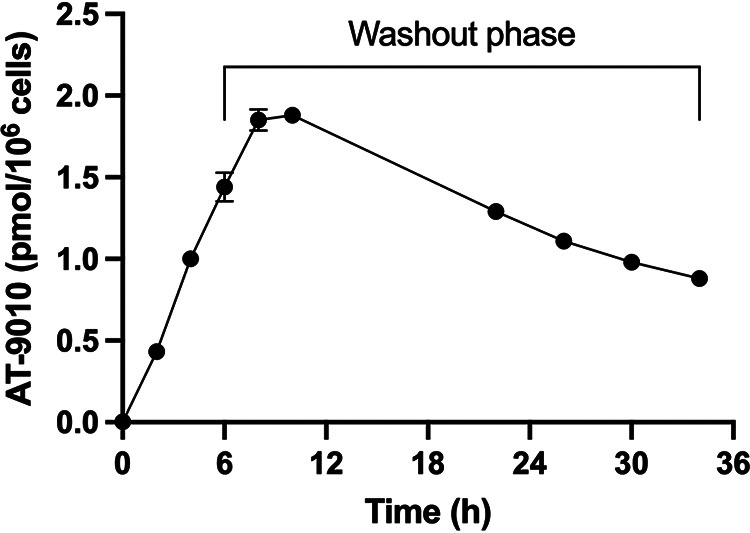
*In vitro* formation of AT-9010 in human peripheral blood mononuclear cells. PBMCs were incubated with 10 μM AT-281 for 6 h. The prodrug was then removed, and the cells were rinsed and incubated for another 28 h (washout phase). At each time point, cells (*n* = 3) were collected in ice-cold 60% MeOH and analyzed for AT-9010, the active intracellular TP, using an LC-MS/MS assay as described in Materials and Methods. Data are expressed as means ± standard deviations (SDs).

Fresh nonstimulated human PBMCs treated with 10 μM AT-281 for up to 6 h had a time-dependent increase in AT-9010 concentrations over the entire exposure phase and continued to increase after drug washout, with a mean peak concentration of 1.88 ± 0.01 pmol/10^6^ cells at 10 h after initiation of exposure (Fig. 2). When AT-281 was removed for 28 h (washout phase), levels of AT-9010 in the PBMCs decreased time dependently (Fig. 2), with an estimated *t*_1/2_ of 21.6 h.

### AT-9010 disrupts RNA elongation by DENV NS5.

**FIG 3:**
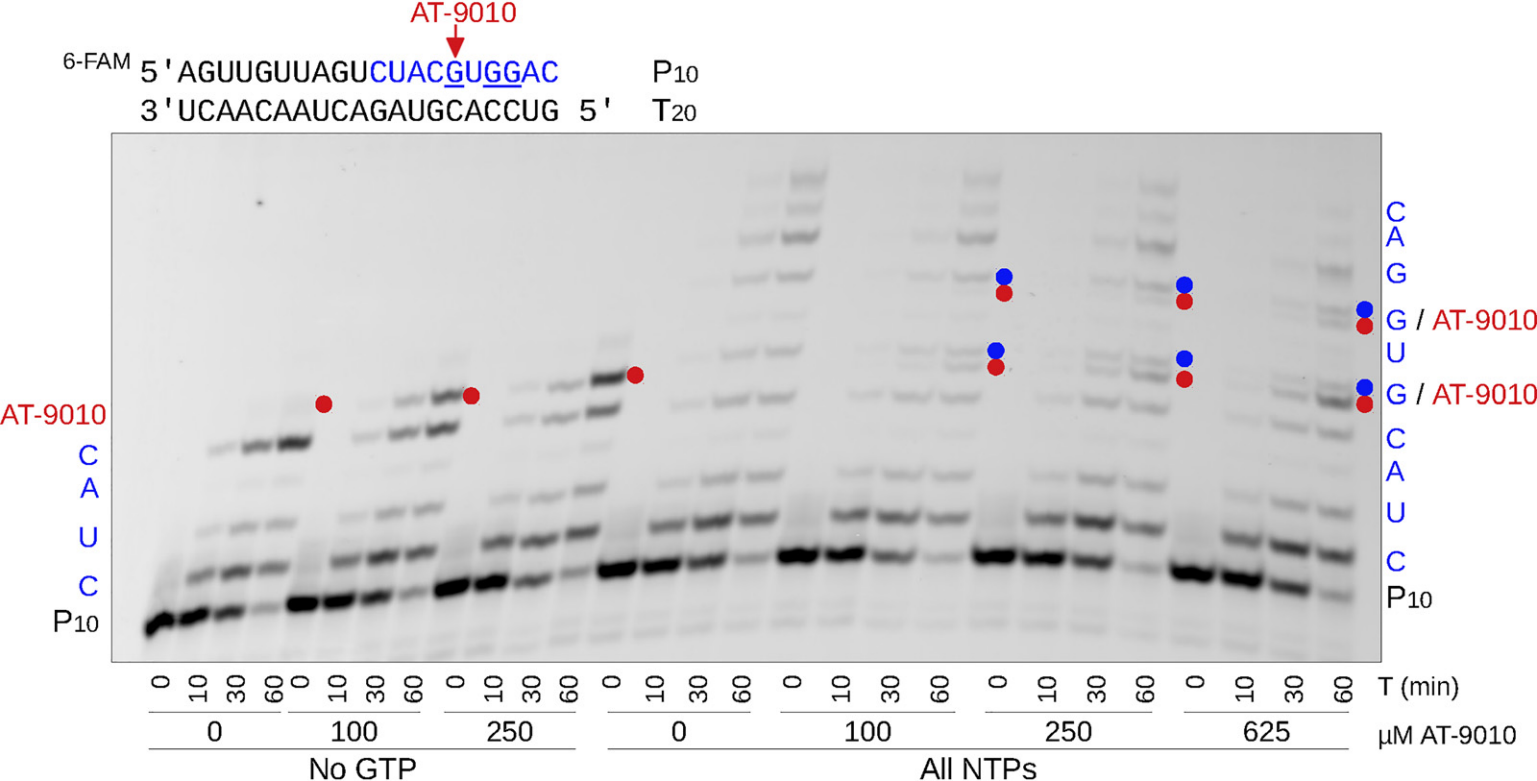
Incorporation of AT-9010 in the presence and absence of GTP. The left half of the gel shows the time course of elongation of P_10_ primer along a T_20_ RNA template by the DENV2 NS5 protein with ATP, UTP, and CTP (100 μM each) and increasing concentrations of AT-9010 (0 to 250 μM). The right half of the gel shows elongation in the presence of all four NTPs (100 μM) in competition with AT-9010 (100 to 625 μM). The AT-9010 incorporation products at +5 and +7 positions are shown with red dots, while incorporation of the native GTP at the same positions are shown with blue dots.

To confirm the mechanism of action and antiviral target of AT-9010, we ran incorporation and elongation assays using the full-length DENV NS5 protein (serotype 2) and an annealed primer-template RNA pair mimicking the 3′ end of the DENV2 genome. In the absence of GTP, AT-9010 was readily incorporated as a substitute at the +5 position of the RNA primer (Fig. 3, left side of gel). Despite the presence of the next correct nucleotide (UTP), no further extension was observed, indicating that AT-9010 causes immediate chain termination of RNA synthesis irrespective of the presence of its 3′ OH group. In the presence of all four nucleoside triphosphates (NTPs), AT-9010 competed with GTP for incorporation at +5 and +7 positions (Fig. 3, right side of gel). In fact, the incorporation of GTP and AT-9010 at the same positions was differentiated on the gel, and the discrimination factor (preference for GTP > AT-9010, as judged by band product intensities) was calculated to be 12.6 ± 4.3. Comparison of incorporation with the obligate chain terminator 3′-dGTP showed an equivalent profile, supporting chain termination as the mechanism of action of AT-9010 (see Fig. S2).

### AT-752 demonstrates favorable pharmacokinetics in preclinical species.

**FIG 4:**
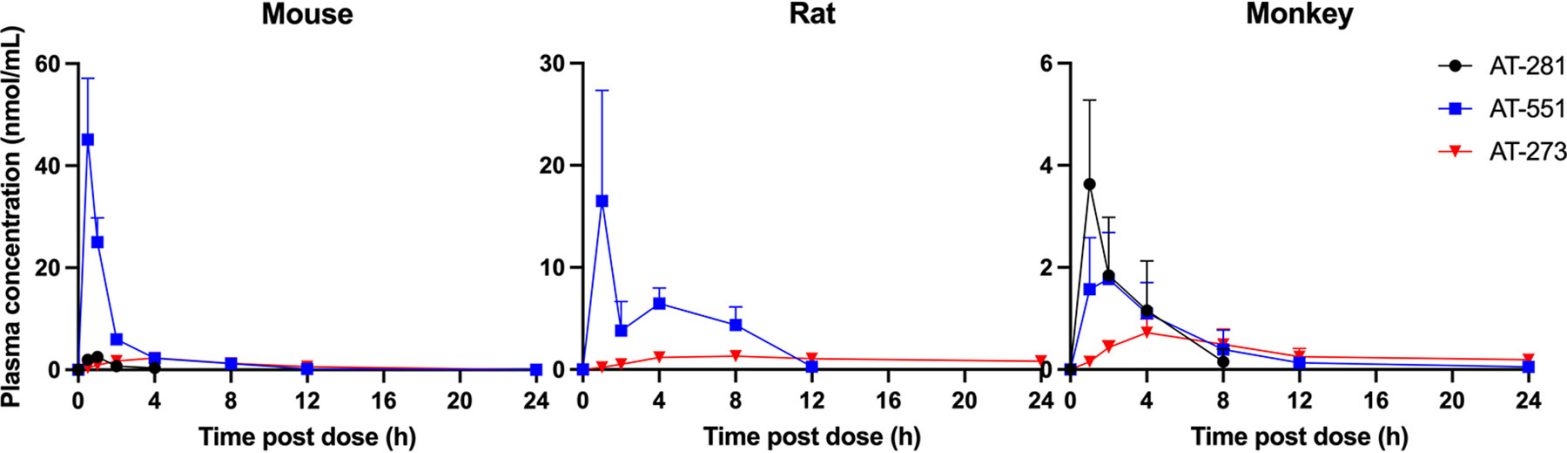
Plasma profiles of AT-281 and its metabolites in mice, rats, and monkeys after oral doses of AT-752. Male CD-1 mice, Sprague Dawley rats, and cynomolgus monkeys were administered 420, 300, and 300 mg/kg AT-752, respectively. Blood samples were collected up to 24 h postdose, and plasma was separated and analyzed for AT-281, AT-551, and AT-273 by LC-MS/MS. Data are expressed as means ± SDs (*n* = 3 to 5 per time point).

The plasma pharmacokinetics (PK) of AT-281 and its metabolites AT-551, the intermediate prodrug, and AT-273, the plasma surrogate for intracellular levels of the active triphosphate metabolite AT-9010, were determined in CD-1 mice, Sprague Dawley rats, and cynomolgus monkeys after single oral doses of AT-752 at 420, 300, and 300 mg/kg body weight, respectively (Table 3). In rodents, the parent prodrug, AT-281, was quickly metabolized with the rapid appearance in plasma of its metabolites, AT-551 and AT-273 (Fig. 4). For rats, the plasma concentration of AT-281 was below the limit of quantitation at 1 h, the earliest time point measured, which is consistent with the metabolic instability of AT-281 in rat plasma reported above. In monkeys, AT-281 was present for more than 4 h in plasma (Fig. 4), with a maximum concentration of drug in serum (*C*_max_) of 3.7 ± 1.5 nmol/ml at 1 h. The prodrug was converted to the plasma metabolites AT-551 and AT-273, with PK comparable to previously reported results of its congener AT-511 (15).

**TABLE 3:** Pharmacokinetic parameters following administration of a single oral dose of AT-752^*a*^

Metabolite	Species	Dose (mg/kg)	*C*_max_^*b*^ (nmol/ml)	*T*_max_^*c*^ (h)	AUC_0–24_^*d*^ (nmol · h/ml)
AT-281	Mouse	420	2.6 ± 0.3	0.8 ± 0.3	4.2 ± 0.5
	Rat	300	ND^*e*^	ND	ND
	Monkey	300	3.7 ± 1.5	1.0 ± 0.0	9.5 ± 3.7
AT-551	Mouse	420	45.1 ± 12.0	0.5 ± 0.0	58.7 ± 13.3
	Rat	300	16.5 ± 10.8	1.0 ± 0.0	56.5
	Monkey	300	2.0 ± 0.8	2.0 ± 0.0	9.7 ± 2.2
AT-273	Mouse	420	2.3 ± 0.7	4.0 ± 0.0	18.8 ± 6.0
	Rat	300	1.3 ± 0.3	8.0 ± 0.0	22.9
	Monkey	300	0.8 ± 0.2	4.0 ± 0.0	7.8 ± 2.4

a Blood samples were collected up to 24 h postdose, plasma was separated, and concentrations of the free base AT-281 and its metabolites (see Fig. 1 for the putative pathway) were measured by LC-MS/MS. Data are expressed as means ± SDs (*n* = 3 to 5), except for rat AUC data, where there is no SD because composite sampling was used, resulting in pooled concentration data for this parameter.

b *C*_max_, maximum concentration across the time points measured.

c *T*_max_, time at which *C*_max_ was observed.

d AUC_0–24_, area under the curve, from 0 h to the 24 h time point.

e ND, not determined because concentration was below the limit of quantitation.

### The active metabolite AT-9010 is formed in PBMCs *in vivo*.

**FIG 5:**
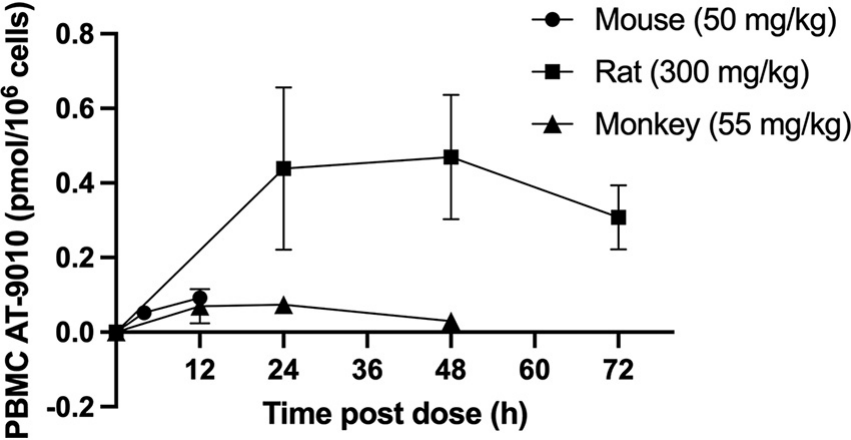
AT-9010 in mouse, rat, and monkey PBMCs after single oral doses of AT-281. Mice and rats were administered an oral dose of AT-281, and blood was collected at 4 and 12 h (mice) and at 24, 48, and 72 h (rats). Monkeys were given an oral dose of AT-752 equivalent to AT-281 at 55 mg/kg, and blood was collected at 12, 24, and 48 h. PBMCs were isolated and AT-9010 was extracted and quantitated using LC-MS/MS as described in Materials and Methods. Mouse PBMCs were pooled from 3 animals per time point; rat and monkey data are expressed as means ± SDs (*n* = 2 or 3 per time point).

Concentrations of AT-9010 in PBMCs isolated from mice and rats administered single oral doses of AT-281 at 50 and 300 mg/kg and monkeys after a single oral dose of AT-752 equivalent to 55 mg/kg AT-281 were measured at various time points postdose (Fig. 5). For comparable doses, similar concentrations of AT-9010 were observed in mice and monkeys at 12 h (0.092 and 0.070 ± 0.046 pmol/10^6^ cells). At a 6-fold higher dose, PBMCs from rats at 24 h exhibited 5- to 6-fold more AT-9010 (0.439 ± 0.218 pmol/10^6^ cells) than the 12-h values observed in mice and monkeys, suggesting a similar extent of active TP formation in PBMCs across all three species, despite the very low exposure to AT-281 in the systemic circulation of rats and, to a lesser extent, mice. When mice were given a loading dose of 500 mg/kg AT-281, followed by a second dose of 250 mg/kg 4 h later, the concentrations of AT-9010 in PBMCs at 4 and 12 h after the second dose were 0.297 and 0.322 pmol/10^6^ cells, respectively (not shown).

To test the tolerability of AT-281 and determine steady-state concentrations of the active TP in PBMCs, mice were given a loading dose of 1,000 mg/kg and, starting 4 h later, dosed twice a day (12 h apart) at 500 mg/kg for 3 days. The concentrations of AT-9010 in pooled (*n* = 3) PBMCs 12 h after the first and sixth 500-mg/kg dose were 0.421 and 0.575 pmol/10^6^ cells, respectively, while the concentrations of plasma AT-273 were 0.67 ± 0.12 and 1.35 ± 0.86 nmol/ml, respectively, for the same time points. There were no adverse clinical signs observed in the mice, and so the multiple doses of the prodrug were well tolerated.

### AT-752 reduces viremia and improves survival of AG129 mice after DENV2 D2Y98P infection.

**FIG 6:**
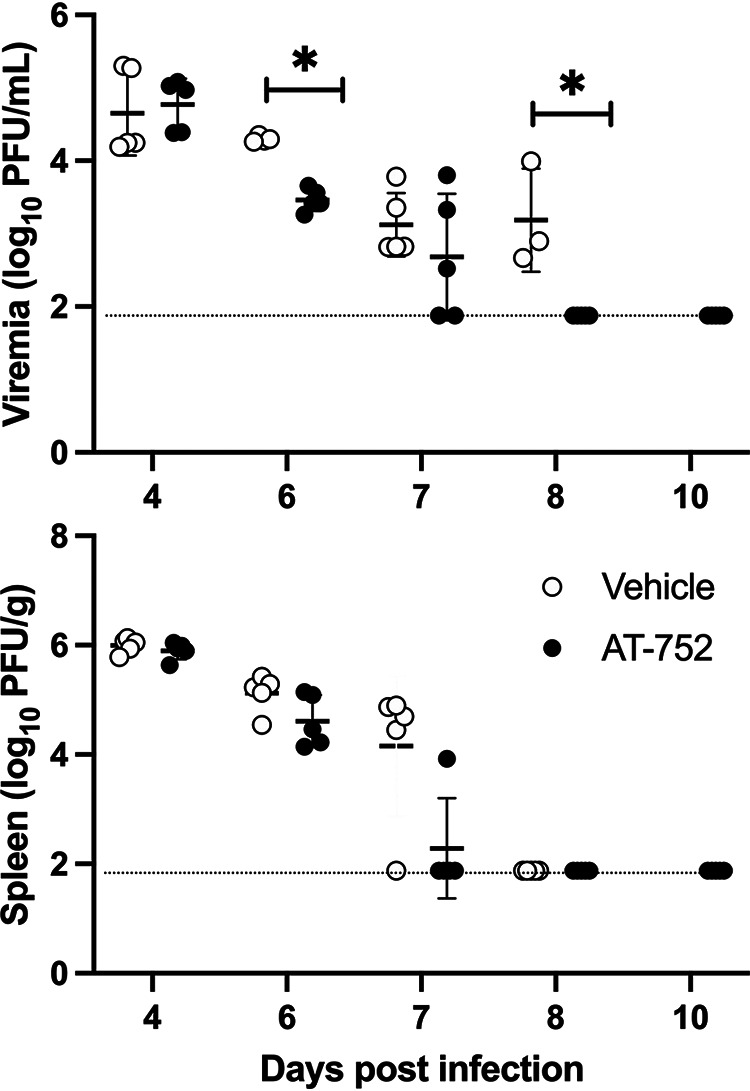
Viremia and spleen viral load in DENV-infected mice treated with AT-752. AG129 mice challenged with DENV2 were treated orally with vehicle (control) or AT-752 (1,000 mg/kg loading dose 4 h prior to challenge, followed by BID doses of 500 mg/kg for 7 consecutive days, starting 1 h postinoculation). Blood and spleen samples were collected on days 4, 6, 7, 8, and 10 postinfection and assessed for viral load by a plaque assay described in Materials and Methods. Dotted lines indicate the limit of detection; data are expressed as means ± SDs. *, *P* ≤ 0.01 by *t* test at each time point.

To test the efficacy of AT-752 against DENV infection, AG129 mice were subcutaneously inoculated with DENV2 D2Y98P, a non-mouse-adapted strain that follows a similar disease kinetic as described in humans (19). The prodrug was orally administered as a loading dose (1,000 mg/kg) 4 h prior to viral challenge and afterwards as twice a day (BID) doses (500 mg/kg) for 7 consecutive days, beginning 1 h postinfection (pi). Viremia and spleen viral loads, the primary endpoints, were evaluated at days 4, 6, 7, 8, and 10 pi. Viremia (Fig. 6) in the AT-752-treated group was significantly lower than in the vehicle only group on day 6 pi (*P* = 0.000014 by *t* test) and was cleared in all treated animals by day 8 pi, whereas virus was still measurable in all surviving controls (*P* = 0.0045 by *t* test). Although AT-752 treatment reduced the spleen viral load by day 7 pi compared to that in the control group (Fig. 6), this change was not statistically different between the groups. This result is likely because the virus is eventually cleared from the spleen in this mouse model of disease.

**FIG 7:**
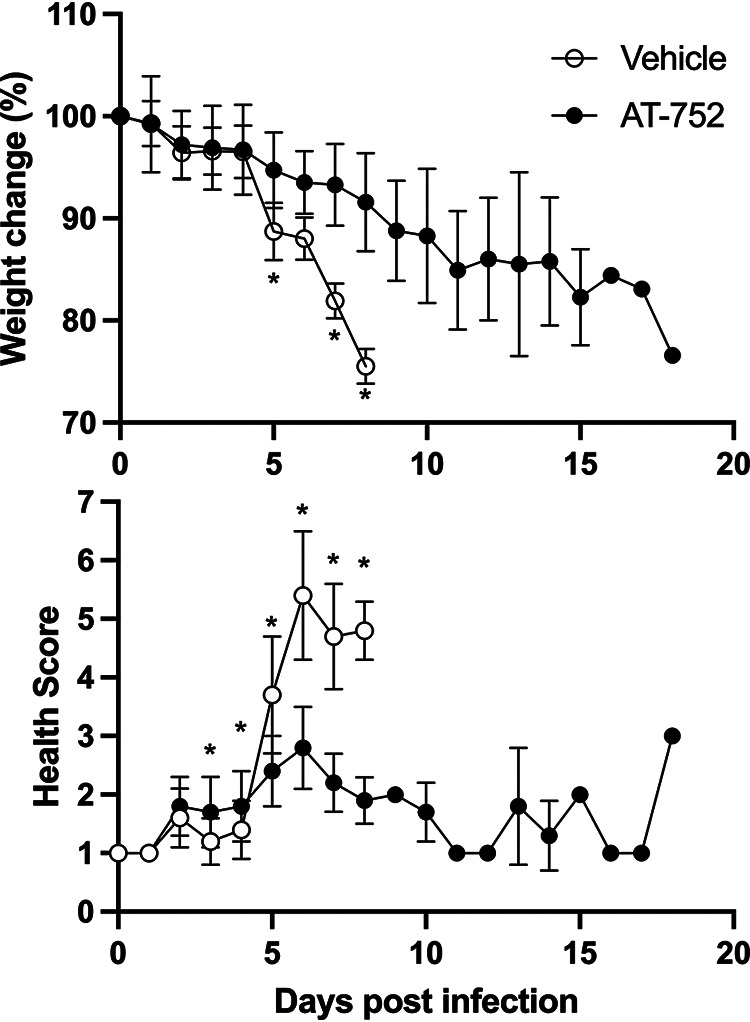
Weight change and health scores of DENV-infected mice treated with AT-752. AG129 mice challenged with DENV2 were orally administered vehicle (control) or AT-752 (loading dose of 1,000 mg/kg 4 h prior to challenge, followed by BID doses of 500 mg/kg for 7 consecutive days starting 1 h postinoculation). Body weights and health scores were recorded daily for the duration of the study. Weight was compared to that at day 0 (baseline) and presented as percent change. Data are expressed as means ± SDs. *, *P* ≤ 0.01 by *t* test at each time point.

**FIG 8:**
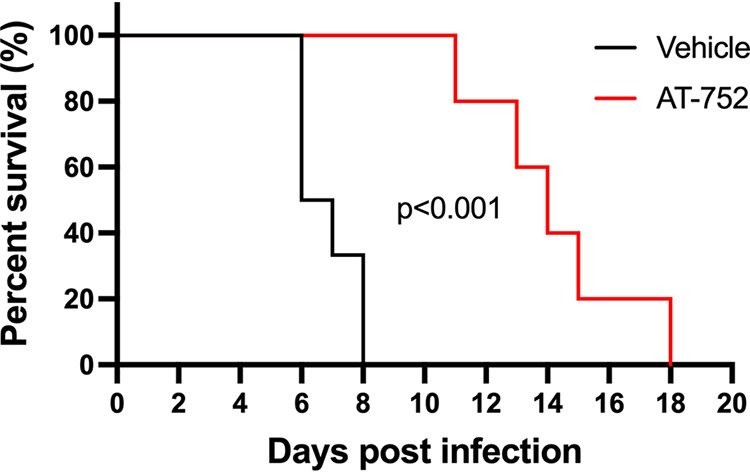
Kaplan-Meier survival curves of DENV-infected mice treated with AT-752. AG129 mice challenged with DENV2 were administered vehicle (control) or AT-752 (loading dose of 1,000 mg/kg 4 h prior to challenge, followed by BID doses of 500 mg/kg for 7 consecutive days starting 1 h postinoculation). Percent survival was calculated up to 20 days postinfection, excluding mice sacrificed as scheduled for collection of blood and spleen samples. The treated group was significantly different from control, *P* ≤ 0.001 by the Mantel-Cox log rank test.

Mice were evaluated daily for weight change, appearance, mobility, and alertness and euthanized when 20% weight loss or a health score of >5 was reached. There were notable differences in body weight between the treated and vehicle control groups at days 4 to day 8 pi (*P* < 0.001 by *t* test). No vehicle-treated mice survived beyond day 8 pi. Animals treated with AT-752 had significantly reduced weight loss compared to vehicle control mice on days 5, 7, and 8 pi (Fig. 7) (*P* < 0.001 by *t* test). In addition, mice treated with AT-752 had significantly better health scores than the vehicle control group on day 4 to day 9 pi (Fig. 7) (*P* < 0.001 by *t* test). The resulting mortality data, presented as Kaplan-Meier survival curves (Fig. 8), demonstrate that AT-752 significantly improved the survival of the infected mice compared to that of the vehicle control group (*P* = 0.0004, Mantel-Cox log rank test). All mice in the control group not sacrificed as scheduled for collection of blood and spleen samples died or were euthanized on days 5 to 8 pi. In contrast, those animals treated with AT-752 survived to between days 11 and 18 pi.

## DISCUSSION

Infection from DENV, the most prevalent mosquito-borne virus that causes human disease, is on the rise throughout the world, possibly aided by anthropogenic global warming, leaving millions vulnerable to developing dengue hemorrhagic fever or severe dengue, resulting in significant mortality. Currently, there are no DAAs or broadly indicated vaccines for DENV, but given our previous work developing a safe and efficacious oral DAA that targets the HCV RdRp critical for viral replication (14, 15), we took aim at the same objective for DENV. The DENV and HCV RdRp active sites are homologous (16) and highly conserved in *Flaviviridae*, potentially allowing for a single agent to be active against all four serotypes of DENV. This would be important since, according to the WHO (https://www.who.int/news-room/fact-sheets/detail/dengue-and-severe-dengue), recovery from one serotype does not give immunity to the other serotypes, and subsequent infection by other serotypes increases the risk of severe disease and death. Moreover, while diagnosis of dengue virus infection can be straightforward and rapid, distinguishing the serotype requires additional instrumentation, usually in a laboratory setting. Thus, the ideal treatment for dengue fever should possess pan-serotypic potencies (10). To that end, we have demonstrated that AT-752, an orally available double prodrug of a guanosine nucleotide analog, has potent *in vitro* activity against DENV2 and DENV3. The free base of AT-752, like its congener AT-511 (15), also inhibits HCV. With an identical metabolic disposition once the first stable metabolite, AT-551, is formed, AT-752 is likely to have a safety profile similar to that of AT-527 and is worthy of clinical development, unlike other nucleoside analogs with *in vitro* activity against DENV (10, 20). Indeed, initial preclinical toxicology studies of AT-752 in rats and monkeys have indicated no safety concerns at doses up to 1,000 mg/day for 14 days (S. S. Good, unpublished data).

In this mouse model of dengue infection, it is somewhat surprising that a viral replication inhibitor such as AT-752 did not demonstrate a difference in viremia between treated and control animals on day 4 postinfection (pi). We are unaware of any other replication inhibitors that are effective in this animal model with the D2Y98P virus strain with which this result can be compared; thus, this result may be a typical characteristic of this model. Nevertheless, the statistically significant differences in viremia observed on days 6 and 8 pi, combined with the significant effects on prevention of weight loss and deterioration of health score on days 3 through 8 (last day of surviving controls) and on the highly significant prolongation of survival, attest to the efficacy of AT-752.

NS5 is the largest and most highly conserved of the nonstructural proteins among the DENV serotypes and within the *Flavivirus* genus and contains both methyltransferase and RdRp activities, the latter being the virus-specific enzyme which catalyzes the replication of viral RNA (10, 21). Using the full-length DENV2 NS5 with a specific RNA primer/template to create an active elongation complex, we showed that AT-9010 acted as a GTP analog and was incorporated into RNA by the RdRp, immediately terminating synthesis. Similar results have been reported for the severe acute respiratory syndrome coronavirus 1 (SARS-CoV-1) and SARS-CoV-2 polymerases, with structural analysis showing the 2′-fluoro-2′-C-methyl ribose modification of AT-9010 impacts several important interactions with palm domain residues required for proper alignment of the incoming NTP, subsequently preventing catalysis (22).

The cytosine analog balapiravir appeared to be efficacious against DENV *in vitro*, but when tested in patients infected with DENV, it failed to reduce viremia (12). This may not be the case for AT-752, since the enzymes putatively involved in its activation pathway (Fig. 1) are different from those that activate balapiravir (23, 24). Chen et al. reported that when PBMCs, one of the major cell types targeted for viral replication, were infected with DENV, they were less efficient in converting balapiravir to its intracellular active TP, resulting in decreased antiviral potency *in vivo* (24). However, the nucleoside backbone was important, since NITD008, an adenosine-based inhibitor, was much less affected. AT-752 has a purine backbone too, being a guanosine prodrug, and here, we used a dengue mouse model to show that mice infected with DENV and treated with AT-752 had decreased viremia and increased survival (Fig. 8) compared to the control group that did not receive the prodrug. We have shown that despite the high expression of esterase activities in the plasma of rodent species (25) that can quickly break down AT-281, after oral administration, metabolism of the prodrug and conversion to AT-9010 in the PBMCs of rats and mice appear to be similar to those in PBMCs of monkeys, a species more analogous to humans in which AT-281 is more stable in blood. This suggests that the intermediate prodrug AT-551 may also serve as a source of the TP in these white blood cells. The elevated levels of AT-9010 maintained with BID dosing in mice, combined with the 22-h half-life of the active TP observed in human PBMCs *in vitro* and the efficacy of AT-752 in the *in vivo* mouse model of DENV infection, suggest that therapeutic steady-state levels of AT-9010 can be achieved and maintained with repeated doses of AT-752 in patients with DENV. Thus, AT-752 is a promising compound for clinical development in the treatment of dengue virus and possibly other flavivirus infections.

## MATERIALS AND METHODS

### Cell lines, viruses, and test compounds.

Huh-7 (human liver carcinoma; AcceGen Biotechnology, Fairfield, NJ) cells were maintained in Dulbecco’s modified Eagle medium (DMEM) supplemented with 10% fetal bovine serum (FBS), 100 μg/ml penicillin, and 100 μg/ml streptomycin (Lonza, Walkersville, MD). Vero 76 cells (American Type Culture Collection [ATCC], Manassas, VA) used for the virus yield reduction assay were maintained similarly. BHK-21 (baby hamster kidney; ATCC, Manassas, VA) cells were maintained in minimum essential medium with Earle’s salts (EMEM) containing 1 mM sodium pyruvate and 25 μg/ml kanamycin and supplemented with 10% FBS. All cell cultures were maintained at 37°C in an atmosphere of 5% CO_2_ and ≥95% humidity. Infections were performed in EMEM supplemented with 5% FBS and 50 μg/ml gentamicin.

The dengue viruses (DENV2 NGC and DENV3 H87) were obtained from ATCC (Manassas, VA), and DENV2 used in the human PBMC and BHK-21 assays was a clinical isolate. Japanese encephalitis (JEV SA-14), West Nile (WN02 Kern 515), yellow fever (YFV 17D), and zika (ZIKV MR766) viruses were obtained from the University of Texas Medical Branch (Galveston, TX), while the Powassan (POWV Spooner and LB strains) and Usutu (USUV TC-508) viruses were from the World Reference Center for Emerging Viruses and Arboviruses at the University of Texas Medical Branch. For studies using fresh human PBMCs, cells from a single male donor (lot LS-88-45477C) were obtained from BioIVT (Westbury, NY) or were isolated from blood collected from healthy donors using Ficoll-Paque (26, 27). The DENV2 studies in PBMCs and BHK-21 cells were completed at Università degli Studi di Cagliari (Monserrato, Italy). The other virus studies for efficacy and specificity were conducted at Utah State University (Logan, UT) and ImQuest BioSciences (Frederick, MD). Protocols at the latter laboratory for the different RNA and DNA viruses—HCV, HIV-1, influenza A and B virus, respiratory syncytial virus (RSV), rhinovirus, herpes simplex 1, and adenovirus—have been previously described (15). AT-752 and its free base AT-281 were synthesized by a stereospecific process and, along with its plasma metabolite AT-273, were prepared for Atea Pharmaceuticals by Topharman Shanghai Co., Ltd., Shanghai, China. AT-9010 and the TP internal standards used to quantify AT-9010 were synthesized by NuBlocks (Oceanside, CA). Stock solutions were prepared in dimethyl sulfoxide (DMSO) and stored at −20°C. Frozen plasma, human and from Sprague Dawley rat (BioreclamationIVT, Westbury, NY) and from cynomolgus monkey (Suzhou Xishan, China), was purchased to test the stability of AT-281 at 37°C.

### Flavivirus infection and treatment of Huh-7 cells.

The antiviral activity of AT-281 was evaluated against flaviviruses DENV2 NGC, DENV3, JEV, POWV, USUV, WNV, YFV, and ZIKV by using a neutral red dye uptake assay to determine inhibition of virus-induced and compound-induced CPE and by using a virus yield reduction (VYR) assay as a second independent determination of the inhibition of viral replication.

### Neutral red assay.

Test compounds were dissolved in DMSO at a concentration of 10 mg/ml and serially diluted using eight half-log dilutions so that the highest test concentration was 100 μg/ml (172 μM). Each dilution was added to 5 wells of a 96-well plate with Huh-7 cells at 80% to 100% confluence. Three wells of each dilution were infected with virus, and two wells remained uninfected as toxicity controls. Six untreated wells were infected as virus controls, and six untreated wells were left uninfected to use as virus controls. Viruses were diluted to achieve a multiplicity of infection (MOI) of approximately 0.001 CCID_50_ (50% cell culture infectious dose) per cell (0.037 and 0.028 CCID_50_ for POWV LB strain and USUV, respectively). Plates were incubated at 37°C in a humidified atmosphere containing 5% CO_2_. On day 3 (ZIKV, Eastern equine encephalitis virus [EEEV], and POWV Spooner), day 4 (chikungunya virus [CHIKV]), day 5 (YFV, Rift Valley fever virus [RVFV], Middle East respiratory syndrome [MERS] virus, POWV LB, and USUV), or day 6 (WNV, JEV, DENV2 NGC, and DENV3) postinfection, when untreated virus control wells reached maximum CPE, the plates were stained with neutral red dye for approximately 2 h (±15 min). Supernatant dye was removed, wells were rinsed with phosphate-buffered saline (PBS), and the incorporated dye was extracted in 50:50 Sorensen citrate buffer-ethanol for >30 min. The optical density was read on a spectrophotometer at 540 nm and converted to percentage of controls. The concentrations of test compound required to prevent virus-induced CPE by 50% (EC_50_) and to cause 50% cell death in the absence of virus (CC_50_) were calculated (28, 29). The selective index is the CC_50_ divided by EC_50_ except where indicated. Selectivity index (SI) values between 0 and 3.9 indicate inactive compounds, SI of 4 to 9.9 indicates minimally active compounds, SI of 10 to 49.9 indicates moderately active compounds, and SI values >50 indicate highly active compounds. Compounds with SI values >100 are indistinguishable from one another.

### Virus yield reduction assay.

As previously published (30), Vero 76 cells were seeded in 96-well plates and grown overnight (37°C) to confluence. A sample of the supernatant fluid from each compound concentration was collected on day 3 (day 4 for POWV and day 7 for USUV) postinfection (3 wells pooled) and tested for virus titer using a standard endpoint dilution CCID_50_ assay and titer calculations using the Reed-Muench equation (31). The concentration of compound required to reduce virus yield by 90% (EC_90_) was determined by regression analysis.

### DENV2 infection and treatment of human PBMCs and BHK-21 cells.

AT-281 was dissolved in DMSO at 100 mM and then diluted in growth medium to final concentrations of 100, 20, 4, and 0.8 μM. PBMCs were resuspended in RPMI 1640 medium with 2 mM l-glutamine, 10% FBS, 100 U/ml penicillin, and 100 μg/ml streptomycin (PBMC growth medium) and were subjected to a 3-day activation with phytohemagglutinin (PHA) (5 μg/ml). Following a 1-h infection with DENV2, the cells were seeded in 96-well plates (1 × 10^5^ cells/well) in PBMC growth medium, either in the absence or presence of serial dilutions of AT-281. Similarly, BHK-21 cells were grown to confluence in 96-well plates, and then growth medium was replaced with fresh maintenance medium (growth medium with 1% inactivated FBS in place of 10% FBS) containing serially diluted AT-281 and DENV2 at a multiplicity of infection (MOI) of 0.01. Uninfected cells in the presence of serially diluted compound were used to assess the cytotoxicity of compounds. After a 3-day incubation at 37°C in a humidified 5% CO_2_ atmosphere, cell viability was determined by the 3-(4,5-dimethyl-2-thiazolyl)-2,5-diphenyl-2H-tetrazolium bromide (MTT) method (32). The effective concentrations of test compound required to prevent virus-induced cytopathic effect (CPE) by 50% (EC_50_) and to cause 50% cell death in the absence of virus (CC_50_) were calculated by regression analysis.

### Cardiomyocyte assay.

Cytotoxicity was evaluated in human iPS cardiomyocytes (Cellular Dynamics, Madison, WI) and in primary human bone marrow progenitor cells (Invitrogen, Grand Island, NY). Doxazosin mesylate and AZT, used as positive controls, respectively, were purchased from Sigma-Aldrich. The iPS cardiomyocytes, in medium from Cellular Dynamics, were seeded in 96-well plates precoated with 0.1% gelatin (Sigma) at 1.5 × 10^4^ cells/well in a final volume of 100 μl and incubated at 37°C in 5% CO_2_ for 48 h. The cells were washed with Dulbecco's phosphate-buffered saline (DPBS), and test compounds diluted in the same medium (100 μl) were added to the monolayer in triplicates and incubated at 37°C in 5% CO_2_ for 3 days. Cell viability was measured by staining with CellTiter Glo. The medium was removed from the test plates and replaced with fresh medium (100 μl) and CellTiter Glo reagent (100 μl per well) before incubating at room temperature (RT) for 10 min. The well contents were transferred to a white 96-well plate, and luminescence was measured within 15 min on a Wallac 1450 MicroBeta TriLux liquid scintillation counter.

### Bone marrow progenitor cell assay.

Bone marrow progenitor cells suspended in Iscove’s modified Dulbecco medium containing 15% heat-inactivated FBS, 10% giant cell tumor conditioned medium (bone marrow plus; Sigma), 10 ng/ml recombinant human interleukin 6 (IL-6), 10 ng/ml recombinant human IL-3, 25 ng/ml recombinant human granulocyte-macrophage colony-stimulating factor (GM-CSF; R&D systems), and a final concentration of 1% methylcellulose were added to 6-well plates (1 × 10^5^ cells/well) in a volume of 900 μl. Test compounds (100 μl) at 10 times the high test concentration were added to each well in triplicates and incubated at 37°C in 5% CO_2_ for 14 days, and colonies (greater than 30 cells) were counted.

### Formation of AT-9010 *in vitro*.

Upon receipt, fresh human PBMCs (single male donor, lot LS-88-45477C) were centrifuged at 400 × *g* for 5 min. The resulting cell pellet was suspended in 20 ml of warm PBMC growth medium. The cell density of the cell suspension was counted using an automated cell counter (Cellometer K2; Nexcelom) after staining with trypan blue and adjusted to 2 × 10^6^ viable cells/ml. The human PBMC suspension was transferred to 24-well tissue culture treated plates at 0.5 ml/well (1 × 10^6^ cells) and cultured in a humidified incubator maintained at 37°C and 5% CO_2_ for 20 to 24 h. Then, AT-281 stock solution was spiked into each well to achieve the final concentration of 10 μM, and the cells were incubated for 0, 2, 4, and 6 h at 37°C and 5% CO_2_ atmosphere. At each time point, samples in triplicates were processed for analysis of AT-9010. For the washout samples, the incubated PBMCs were collected and centrifuged at 400 × *g* for 5 min. The supernatant was discarded, and the cells washed with 0.5 ml medium, resuspended in 0.5 ml medium, and transferred to individual wells of 24-well plates. The human PBMCs were further incubated in a humidified incubator maintained at 37°C and 5% CO_2_ for an additional 0, 2, 4, 16, 20, 24, and 28 h (i.e., 6, 8, 10, 22, 26, 30, and 34 h, respectively, after the initiation of test article exposure). At each time point, triplicate samples were processed for AT-9010 analysis as follows. Samples were transferred into 2-ml tubes and centrifuged at 800 × *g* for 5 min. After aspirating the supernatant, each sample was vortexed after the addition of 0.1 ml water. They were then quenched with 0.3 ml ice-cold 60% methanol (MeOH) and stored at −70°C until analysis by liquid chromatography-tandem mass spectrometry (LC-MS/MS).

### DENV NS5 protein expression and purification.

Full-length DENV NS5 (serotype 2, New Guinea C) was expressed and purified as previously described (21). Briefly, the gene coding for NS5 with an N-terminal 6×His tag was expressed from a pQE30 vector in Escherichia coli NEB Express cells (New England BioLabs, Ipswich, MA) transformed with pRare2-LacI (Novagen, Madison, WI) and induced with 50 μM isopropyl-β-d-thiogalactopyranoside (IPTG) and 2% ethanol (EtOH) until the OD_600_ value reached 0.6. Cells were lysed by sonication, and the NS5 protein was separated using TALON metal-affinity resin slurry (Clontech, Mountain View, CA) according to the manufacturer’s instructions, washed, and eluted. Size exclusion chromatography was carried out as a second purification step, where the protein was loaded onto a Superdex 200 HR 16/20 column (GE Healthcare) and eluted in a buffer containing 50 mM HEPES (pH 7.5), 300 mM NaCl, 10% glycerol, and 1 mM dithiothreitol (DTT).

### Oligonucleotides and nucleotides.

RNA oligonucleotides were purchased from Biomers.net (Ulm/Donau, Germany). A 20-nucleotide (nt) template sequence (T_20_) corresponding to the 3′ end of the DENV2 antigenome was annealed to a 10-nt complementary primer (P_10_) containing a fluorescent 6-carboxyfluorescein (FAM) moiety at the 5′ end. Annealing was performed using a primer-template molar ratio of 1:1.5 in the presence of 110 mM KCl, incubated at 70°C for 10 min, and cooled slowly to room temperature. NTPs were purchased from GE Healthcare (Chicago, IL).

### Nucleotide analogue incorporation assay.

DENV NS5 was preincubated with the annealed P_10_/T_20_ RNA in an assembly buffer containing 20 mM HEPES (pH 7.5), 10% glycerol, 5 mM MgCl_2_, and 5 mM DTT for 10 min at 30°C to create an active RNA elongation complex. Reactions were started by adding AT-9010 with either all four NTPs or UTP, ATP, and CTP only. Final concentrations were 0.5 μM NS5, 0.25 μM P_10_/T_20_, 100 μM each NTP, and between 10 and 625 μM AT-9010 in a final buffer containing 20 mM HEPES (pH 7.5), 15% glycerol, 5 mM MgCl_2_, and 5 mM DTT. Reactions were quenched at the designated time points in 2× volume FBD stop solution (formamide, 10 mM EDTA) and analyzed on 20% acrylamide-bisacrylamide (19:1) 7 M urea sequencing gels. RNA products were visualized using a Typhoon FluorImager and analyzed using ImageQuant software.

### Animal welfare.

The studies using CD-1 mice, Sprague Dawley rats, and cynomolgus monkeys were conducted at WuXi AppTec (Suzhou, China) in strict compliance with AAALAC International and NIH guidelines and the People’s Republic of China, Ministry of Science and Technology, Regulations for the Administration of Affairs Concerning Experimental Animals, 2017. Protocols were reviewed and approved by WuXi AppTec’s IACUC prior to study initiation, and all animals were assessed by the WuXi AppTec veterinary staff throughout the studies. All animals were housed in rooms with controlled temperature (18 to 26°C), relative humidity (40% to 70%), and light cycle (12 h artificial light and 12 h dark), with 100% airflow. They were provided with manipulatives/enrichment toys. The AG129 mouse study described herein was conducted at IBT Bioservices (Rockville, MD) in strict compliance with USDA Animal Welfare Act and in accordance with the PHS and NIH Policy of Humane Care and Use of Laboratory Animals and the Guide for the Care and Use of Laboratory Animals, National Research Council–ILAR, revised 2011. The study was conducted in full compliance with protocols that were reviewed and approved by IBT’s Institutional Animal Care and Use Committee (IACUC) prior to study initiation, and mice were assessed and monitored throughout the study by members of the IBT veterinary staff in accordance with PHS Policy and the USDA.

### PK studies and *in vivo* formation of AT-9010 in mice, rats, and monkeys.

Male naive CD-1 mice (Shanghai Sippr BK Laboratory Animals Co. Ltd., China) were administered a dose of AT-752 at 420 mg/kg body weight in 40% polyethylene glycol 400 (PEG 400), 10% Solutol HS15, 50% 100 mM citrate buffer, pH 4.5 (vol/vol), by oral gavage. Blood samples (*n* = 3) were collected at 0.5, 1, 2, 4, 8, 12, and 24 h postdose, and plasma separated in EDTA and 1 μl dichlorvos (2 mg/ml; stabilizing agent to prevent *in vitro* hydrolysis of the ester moiety of AT-281 by blood esterases) and stored at −70°C. Concentrations of AT-281 and metabolites AT-551 and AT-273 were determined in the plasma by LC-MS/MS. For PBMCs, six male naive CD-1 mice were administered a dose of AT-752 (at an AT-281 equivalent dose of 50 mg/kg) in 60% PEG 400 by oral gavage. Blood samples were collected, in groups of three, at 4 and 12 h postdose, PBMCs were isolated, and concentrations of AT-9010 were measured by LC-MS/MS.

Male naive Sprague Dawley rats (Beijing Vital River Laboratory Animals Co. Ltd., China) were administered a dose of AT-752 at 300 mg/kg in 40% PEG 400, 10% Solutol HS15, 50% 100 mM citrate buffer, pH 4.5 (vol/vol), by oral gavage. Blood samples (*n* = 4) were collected at 1, 2, 4, 8, 12 and 24 h postdose, plasma separated and stored as described above. Concentrations of AT-281 and metabolites AT-551 and AT-273 were determined by LC-MS/MS. For PBMCs, nine male naive Sprague Dawley rats were administered a dose of AT-752 (at an AT-281 equivalent dose of 300 mg/kg) by oral gavage. Blood samples were collected, in groups of three, at 24, 48, and 72 h postdose, PBMCs were isolated, and AT-9010 concentrations were determined by LC/MS/MS.

Male non-naive cynomolgus monkeys (Hainan Jingang Laboratory Animal Co. Ltd., China), at least 2 kg body weight, were individually housed during the study in stainless steel mesh cages, provided *ad libitum* access to RO water, fed twice daily (except on the day of dosing when they were fed once) with ∼60 g certified monkey diet each feed (Beijing Vital Keao Feed Co., Ltd., Beijing, China), and given daily treats of fresh fruit. They (*n* = 5) were administered a dose of AT-752 at 300 mg/kg in 40% PEG 400, 10% Solutol HS15, 50% 100 mM citrate buffer, pH 4.5 (vol/vol), by oral gavage, and blood samples (∼0.5 ml) were collected at 1, 2, 4, 8, 12, and 24 h postdose. Plasma was separated at each time point for pharmacokinetics and stored at −70°C. Concentrations of AT-281 and metabolites AT-551 and AT-273 were determined by LC-MS/MS. Separately, three monkeys were given an oral dose of AT-752 as a powder in capsules at 60 mg/kg body weight (AT-281 equivalent dose of 55 mg/kg), and blood samples collected at 12, 24, and 48 h postdose. PBMCs were isolated, and concentrations of AT-9010 were measured by LC-MS/MS. All animals were observed for any unusual or adverse clinical signs just before and immediately after dosing and prior to each blood collection time point, with no such signs noted.

Separately, two mouse studies with multiple dosing were conducted. First, 9 naive CD-1 mice were administered AT-281 in 100 mM citrate buffer, pH 4.5, by oral gavage at 500 mg/kg for the first dose and, 4 h later, 250 mg/kg for the second dose. Blood samples were collected, 3 animals per time point, at 4 (before the second dosing), 8, and 16 h after the first dose, and PBMCs were isolated. Second, 9 naive CD-1 mice were administered AT-281 by oral gavage at 1,000 mg/kg for the first dose, and then 4 h later, they were given twice daily doses of 500 mg/kg 12 h apart for 3 consecutive days. Blood samples were collected, 3 animals per time point, at 16 (received one 500-mg/kg dose), 40 (received three 500-mg/kg doses), and 76 h after the first dose (received six 500-mg/kg doses), and PBMCs were isolated. Concentrations of AT-281 and AT-273 in the plasma and AT-9010 in the PBMCs were determined by LC-MS/MS.

### AG129 mouse study.

Fifty-five female AG129 mice (α, β, and γ interferon knockout), 6 to 8 weeks old, were divided into two groups, with 25 mice in group 1 (vehicle) and 30 mice in group 2. On day 0, all mice were challenged with 1 × 10^5^ PFU of DENV2 D2Y98P by subcutaneous injection. Group 1 animals received vehicle (40% PEG 400 [vol/vol], 10% Solutol HS15 [vol/vol], 50% 100 mM citrate buffer, pH 4.5 ± 0.2 [vol/vol]) by oral gavage BID for 7 consecutive days starting at 1 h pi. Group 2 received a single dose of 1,000 mg/kg of AT-752 4 h prior to challenge, followed by a 500-mg/kg dose 1 h postchallenge (pi). BID dosing of AT-752 at 500 mg/kg was continued for 7 consecutive days. The protocol called for five mice per group to be sacrificed and for terminal sera and spleens to be collected on days 4, 6, 7, 8, and 10 pi. On day 20 pi, any surviving mice in group 2 were sacrificed for terminal sera and spleen collection. Serum samples were stored at −80°C until further analysis. Harvested spleen samples were weighed and flash frozen in a mix of ethanol-dry ice and stored immediately at −80°C. All samples were processed and assayed for viral load via plaque assay. All animals were monitored daily for weight loss, morbidity, mortality, and neurological decay. Mice displaying severe illness as determined by >20% weight loss, a health score of greater than 5 (rating coat appearance, mobility, and alertness), extreme lethargy, and/or paralysis were euthanized and harvested for terminal sera and spleens. Any mouse that was found dead was harvested for spleen collection only.

### Plaque assay for spleen viral load determination.

Spleen samples were homogenized in 250 μl DPBS using a TissueRuptor. Homogenates were then centrifuged, and the supernatants were collected and frozen immediately in two different aliquots until the plaque assay was performed.

On the day prior to the assay, Vero cells were seeded at a density of 1 × 10^5^ cells/well in 1 ml 1% Hi-FBS medium in 24-well tissue culture plates and incubated overnight at 37°C in a 5% CO_2_ atmosphere. The following day, 10-fold serial dilutions (10^−2^ to 10^−5^) of the serum and spleen samples were prepared for titration in triplicates. Cell culture medium from the Vero cells was removed, and 100 μl of MEM medium supplemented with 2 mM l-glutamine and 1× penicillin (Pen)-streptomycin (Strep) was added along with 100 μl of the diluted samples. Cells were incubated for 1 h at 37°C in a 5% CO_2_ atmosphere. Then, 1 ml 0.8% methylcellulose containing 2% FBS supplemented with 2 mM l-glutamine and 1× Pen-Strep was added to the cells without inoculum removal and incubated for an additional 3 days at 37°C in a 5% CO_2_ atmosphere to allow plaque formation. Cells were fixed and permeabilized using a cold 80% ethanol-20% methanol mixture for 30 min at −20°C. Two hundred microliters of anti-dengue monoclonal antibody diluted 1:2,000 in 5% nonfat milk was added to each well and incubated overnight at 4°C. Wells were washed 3 times with 1× DPBS, 200 μl of horseradish peroxidase (HRP)-conjugated goat anti-mouse antibody at 1:2,000 dilution in 5% nonfat milk was added, and the wells incubated for 1 h at room temperature. Viral plaques were resolved and counted using insoluble peroxidase substrate (TrueBlue) and a plaque counter. Data for the spleen viral load were normalized to the spleen weight for each individual mouse.

### LC-MS/MS analysis of AT-281, AT-551, AT-273, and AT-9010.

Plasma samples were prepared for MS analysis by adding internal standards and extracting with 20 volumes chilled acetonitrile (ACN). After vortexing (800 rpm for 10 min) and centrifugation (4,000 rpm, 15 min, 4°C), the supernatants (25 μl) were diluted with an equal volume of H_2_O, mixed, and spun again. After adding internal standards, the PBMC samples (10^6^ cells) were lysed with 300 μl MeOH-H_2_O (70:30 [vol/vol]) and 40 mM dibutylammonium acetate (DBAA), mixed by vortexing. and centrifuged (12,000 rpm, 15 min, 4°C). Aliquots of the supernatants (35 μl) were dried under nitrogen and reconstituted in H_2_O and then mixed and spun again. To measure AT-281 and plasma metabolites AT-551 and AT-273, 4-μl samples were injected onto an Acquity Gemini C_18_ (50 by 4.6 mm), 5 μm ultraperformance liquid chromatography (UPLC) column with a Sciex Triple Quad 6500 mass spectrometer (electrospray ionization [ESI] positive ion, multiple reaction monitoring [MRM] mode). A binary nonlinear gradient with mobile phases A (0.1% formic acid in water) and B (0.1% formic acid in ACN) were used to elute samples at 0.8 ml/min, with a run time of 5 min. For AT-9010 in cells, 8 μl was injected onto an Acquity BEH C_18_ (50 by 2.1 mm), 1.7-μm UPLC column with a API 14000 mass spectrometer (ESI negative ion, MRM mode). A binary nonlinear gradient with mobile phases A (0.001% NH_3_·H_2_O, 0.18 mM DBAA in H_2_O) and B (10 mM *N*,*N*-dimethyl-hexylamine, 3 mM ammonium acetate [NH_4_OAc] in ACN-H_2_O [50:50; vol/vol]) were used to elute samples at 0.5 ml/min, with a run time of 3 min. Standards in 50% MeOH were used for calibration. Ions monitored were *m/z* 538.2/440.0 (AT-9010), 582.3/330.1 (AT-281), 464.2/165.1 (AT-551), and 300.2/152.2 (AT-273). Internal standards (ISS) as described previously (15) were used to correct for variations in recovery.

### PK data analysis.

Plasma concentrations of AT-281, AT-551, and AT-273 were subjected to noncompartmental pharmacokinetic analysis using Phoenix WinNonlin software (version 6.3 or above; Pharsight, Mountain View, CA). The linear/log trapezoidal rule was applied in obtaining the PK parameters.
