# Supplementary material for: Evaluation of AT-752, a Double Prodrug of a Guanosine Nucleotide Analog with In Vitro and In Vivo Activity against Dengue and Other Flaviviruses
Source: Antimicrob Agents Chemother. 2021 Oct 18;65(11):e00988-21. doi: 10.1128/AAC.00988-21 (PMC8522752; doi:10.1128/AAC.00988-21)
Supplement: Supplemental file 1 — Supplemental Figures S1 and S2. Download AAC.00988-21-s0001.pdf, PDF file, 0.2 MB [file aac.00988-21-s0001.pdf]

AT-752 inhibits DENV and other flaviviruses. AT-281, the free base of AT-752, showed similar potent effect in reducing viral titer against seven flaviviruses including 11 different strains tested *in vitro*. The inhibition curves for DENV, WNV and YFV (Fig. S1) demonstrate this consistency.

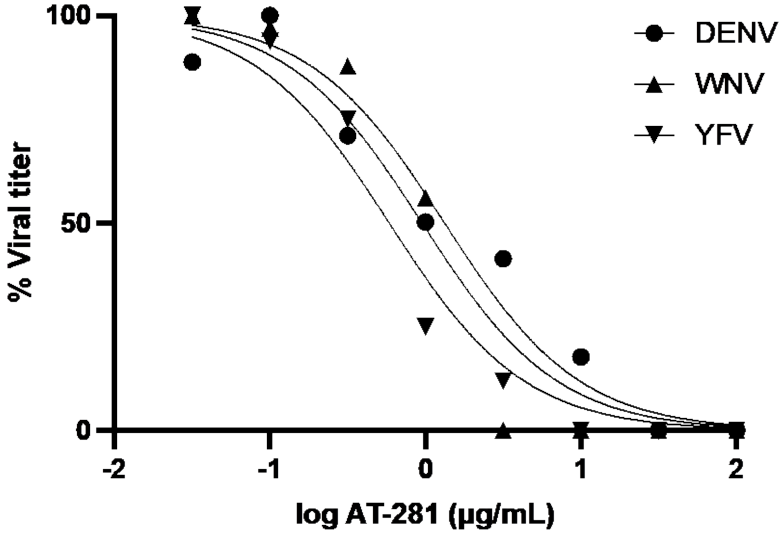

Fig S1: Concentration-dependent inhibition of flaviviruses by AT-281. The activities of AT-281 against dengue-2 virus (DENV), West Nile virus (WNV) and Yellow Fever virus (YFV) were measured using the virus yield reduction (VYR) assay as described in the Methods. The inhibition curves were plotted using GraphPad Prism.

3'-dGTP disrupts RNA elongation by DENV NS5. When we ran incorporation and elongation assays using the full-length DENV NS5 protein (serotype 2) and an annealed primer-template RNA pair mimicking the 3' end of the DENV-2 genome (see method below), we showed that AT-9010 causes immediate chain-termination of RNA synthesis. Comparison of incorporation with the obligate chain terminator 3'-dGTP showed an equivalent profile, supporting chain-termination as the mechanism of action of AT-9010 (Fig S2).

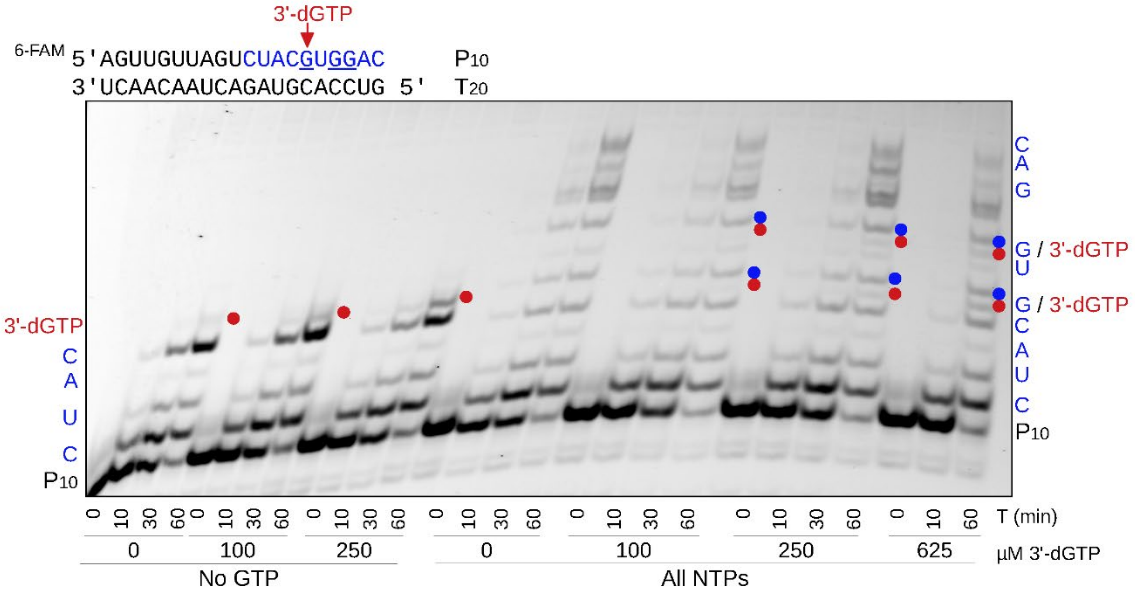

Fig S2: Incorporation of 3'-dGTP in the presence and absence of GTP. The left half of the gel shows the elongation of P_{10} primer by the DENV-2 NS5 protein in the absence of GTP (100 µM ATP, UTP and CTP), with increasing concentrations of 3'-dGTP (0 – 250 µM). The right half of the gel shows elongation in the presence of all four NTPs (100 µM each) in competition with 3'-dGTP (100 – 625 µM). The 3'-dGTP incorporation products at +5 and +7 position are shown with a red dot, while incorporation of the native GTP at the same positions are shown with a blue dot.

Nucleotide analogue incorporation assay. DENV NS5 was preincubated with the annealed P_{10}/T_{20} RNA in an assembly buffer containing 20 mM HEPES pH 7.5, 10% glycerol, 5 mM $MgCl_2$ and 5 mM DTT for 10 min at 30°C to create an active RNA elongation complex. Reactions were started by adding 3'-dGTP with either all four NTPs, or UTP, ATP and CTP only. Final concentrations were 0.5 µM NS5, 0.25 µM P_{10}/T_{20}, 100 µM each NTP, and between 100 – 625 µM 3'-dGTP, in a final buffer containing 20 mM HEPES pH 7.5, 15% glycerol, 5 mM $MgCl_2$ and 5 mM DTT. Reactions were quenched at indicated timepoints in 2X volume FBD stop solution (formamide, 10 mM EDTA) and analysed on 20% acrylamide:bisacrylamide (19:1), 7 M urea sequencing gels. RNA products were visualised using a Typhoon FluorImager and analysed using ImageQuant software.
